# A Comparative Scanning Electron Microscope Investigation of Cleanliness of Root Canals Using Hand K-Flexofiles, Rotary Race and K3 Instruments

**Published:** 2008-10-01

**Authors:** Saeed Rahimi, Vahid Zand, Sharhriar Shahi, Sahar Shakouie, Mohammad Forough Reyhani, Mohammad Mohammadi Khoshro, Pardis Tehranchi

**Affiliations:** 1 *Department of Endodontics, Dental School, Tabriz University of Medical Sciences, Tabriz, Iran*; 2 *Department of Endodontics, Dental School, Shiraz University of Medical Sciences, Shiraz, Iran*; 3 *General Dentist, shiraz, Iran*

**Keywords:** Electron Microscope, Endodontics, Instrumentation, Smear Layer

## Abstract

**INTRODUCTION:** The most important aims of root canal preparation are the removal of vital pulp tissue, remaining necrotic debris and infected dentin, eliminating the bulk of bacteria present in the root canal system. The aim of this study was to compare the cleaning efficacy of hand K-Flexofiles and rotary RaCe and K3 instruments in root canal preparation.

**MATERIALS AND METHODS:** A total of 60 single rooted teeth with maximum curvature of <20º were selected and divided into three groups of 20 teeth each. Canals were prepared with K-Flexofiles, K3 and RaCe instruments using crown down preparation technique, up to size #30. After instrumentation, the root canals were flushed with 5 mL of 2.5 % NaOCl solution. The amount of debris and smear layer was quantified on the basis of Hulsmann method using a scanning electron microscope. The data were statistically analyzed with one-way ANOVA test at a significance level of P<0.05.

**RESULTS:** None of the three groups achieved completely debrided root canals.. In general, K-Flexofiles were able to achieve cleaner canals compared to K3 and RaCe instruments (P<0.05). There were no significant differences between three groups in smear layer removal throughout the root canal walls (P<0.05).

**CONCLUSION:** K-Flexofiles group had less remained debris when compared to K3 and RaCe instruments.

## INTRODUCTION

The major objectives of root canal preparation are elimination of residual pulp tissue, infected dentin and debris and decreased number of microorganism from the root canal system (1,2). The quality of root canal cleaning is evaluated via debris and smear layer removal. Debris contains vital and/or necrotic pulp tissue and dentinal chips that loosely attach to the root canal walls; it is usually infected (3). So debris inhibits removal of root canal's bacteria (4). Smear layer with 1-2 µm of thickness remains on root canal wall after instrumentation (1,5). This layer contains dentinal particles, residual pulpal tissue and bacteria that remain after irrigation sealing the dentinal tubules, which can inhibit the removal of bacteria from the root canal system and therefore root canal seal (5,6).

There are many conflicting reports on the cleaning ability of different hand and rotary instruments (7-11). The past decade has seen the development of nickel-titanium rotary instruments with advanced blade designs; developed to improve the cleaning efficiency during root canal preparation. Rake angle of the cutting blade may affect the cutting and cleaning efficiency of endodontic hand instrument. There are some clues that the flute design of rotary nickel titanium files maybe a key factor for the cleaning efficiently of these instruments. According to a recent report instruments with sharp cutting edges seem to be superior to those having radial lands in cleaning the root canal (12). Positive rake angles will cut more efficiency than neutral or negative rake angles which scrape the inside of the root canal (13). Variable helix angles and pitch is another feature that can improve the removal of the cutting debris formed by instrumentation (14). Once the instrument has cut into dentin, debris needs to evacuate the canal space. Compression occurs when debris is caught between the canal wall and instrument flutes. If the instrument becomes clogged, there will not be any space left for debris to move out of the root canal system. Instruments with consistent helix angle and pitch may allow debris to accumulate, particularly in the coronal part of the file, blocking the escape way of cutting debris (13).

One of the NiTi rotary files is RaCe file (short for reamers with Alternative cutting edges). This file possesses an alternating spiral and has a cutting shank of 8 mm, giving variable helical angles and a variable pitch. A recently produced NiTi rotary file is K3 file. It has a modified radial land with a slightly positive rake angle. The helix flute angle increases from the tip to the handle. Additionally, it has a variable pitch throughout the cutting shank. The manufacturer claims that this design will effectively cut the dentin surface; and dentinal debris can easily be irrigated away (13). However, there is not sufficient data regarding the cleaning ability of these instruments to remove smear layer and debris. The aim of this investigation was to compare the cleaning efficacy after preparation with rotary NiTi K3, RaCe and hand K-Flexofiles.

## MATERIALS AND METHODS

A total of 60 single rooted extracted human teeth with close apex were selected for this *in vitro* study. Standard buccolingual and mesiodistal radiographs were taken for the purpose of appropriate selection of studied samples*.* The teeth with abnormal apex and calcified canal were excluded. Root curvature was determined by using Schneider method and the teeth with<20º curvatures were chosen (15). The teeth were decoronated with a diamond disk (D&Z, Berlin, Germany) and 15mm of root structure was left .Working length was determined by 1 mm less than length of the initial file (size #15) up to the apical foramen. The teeth were then randomly divided into 3 groups as follow (each containing 20 teeth):

***RaCe*** (FKG dentaire, La Chaux-de-Fonds, Switzerland): these instruments were set into rotational speed of 500 RPM with 8:1 reduction handpiece powered by a torque limited electric motor (Novage, Konstanz, Germany). Instrumentation was completed using the crown down technique, according to the manufacture's instruction (16). All canals were sequentially prepared to the apical size #30.

The preparation sequence was:

***1)*** 0.1 tapered size #40 instruments were used to one-third of the working length

***2)*** 0.08 tapered size #35 instruments were used to one-half of the working length

***3)*** 0.06 tapered size #25 instruments were used to two-thirds of the working length

***4)*** 0.04 tapered size #25 instruments were used to full working length

***5)*** 0.02 tapered size# 25 instruments were used to full working length

***6) ***0.02 tapered size #30 instruments were used to full working length

***K3*** (SybronEndo, CA, USA): these instruments were set into rotational speed of 250 rpm with 8:1 reduction handpiece powered by a torque limited electric motor (TCM 3000 Novage, Konstanz, Germany). Instrumentation was completed using the crown down technique (17). All canals were sequentially prepared to the apical size #30 according to the manufacturer's instruction as follow:

***1)*** 0.1 tapered size #25 instruments were used to one-third of the working length

***2)*** 0.08 tapered size #25 instruments were used to one-half of the working length

***3)*** 0.04 tapered size #40 instruments were used to two-thirds of the working length

***4)*** 0.04 tapered size #35 instruments were used to near the working length

***5)*** 0.04 tapered size #30 instruments were used to full working length

***K-Flex Files*** (Dentsply, Maillefer, Ballaigues, Switzerland): hand instrumentation with these instruments was completed using crown down technique. All canals were sequentially prepared to the apical size #30.

**Figure 1:**
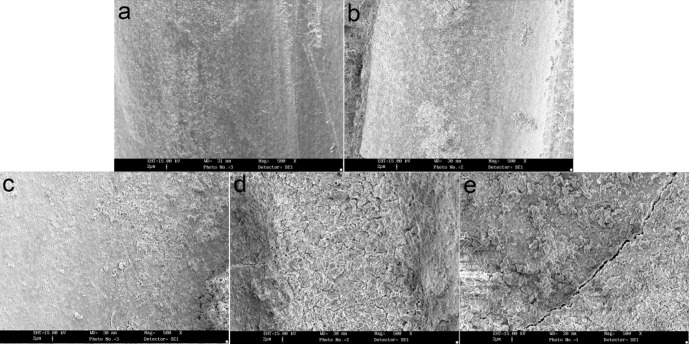
SEM images of debris remained on the canals after preparation (×500 mag) (a) Clean canal wall in the coronal portion of the prepared canal with K-Flexofiles (Score 1),(b) Very small debris particles in the middle portion of the prepared canal with K-Flexofiles (Score 2),(c) SEM image of coronal portion of the prepared canal with Race (Score 3),(d) SEM image of coronal portion of the prepared canal with K3 (Score 4),(e) SEM image of the middle portion of the prepared canal with Race (Score 5)

***1)*** Sequential use of file #45 in coronal parts to #15 in full working length.

***2)*** Sequential use of file #50 in coronal parts to #20 in full working length.

***3)*** Sequential use of file #55 in coronal parts to #25 in full working length.

***4)*** Sequential use of file #60 in coronal part to #30 in full working length.

During instrumentation, the root canals were flushed with 5mL of 2.5 % NaOCl and after instrumentation, 5mL of normal saline was used with a plastic syringe (Yazd Syringe, Yazd, Iran) and 27 gauge needle (Iran needle, Iran) as a final rinse in all groups.

After final rinse with normal saline, two longitudinal grooves were prepared using a No.1 diamond disk on the buccal and lingual aspects of the teeth. The teeth were separated into two halves by a plastic instrument and both halves were prepared for SEM evaluation, and examined under the Leo-440i-SEM (Leo electron microscopy, Cambridge, UK) at ×500 for debris and ×1500 for smear layer evaluation. The cleanliness of each root canal was evaluated in three areas (apical, middle and coronal thirds of the roots) by means of a numerical evaluation scale (3). The canal cleanliness was evaluated by blind observation. The following scheme was used (8):

***Debris:***

*Score 1*: clean canal wall, few debris particles

*Score 2*: few small agglomerations

*Score 3*: many agglomerations, less than 50% of canal wall covered

*Score 4*: more than 50% of the canal wall covered

*Score 5*: complete or nearly complete covering of the canal wall by debris.

***Smear layer:***

*Score 1*: no smear layer, orifice of dentinal tubules patent

*Score 2*: small amount of smear layer, some open dentinal tubules

*Score 3*: homogenous smear layer along almost the entire canal wall, only very few open dentinal tubules

*Score 4*: the entire root canal wall covered with a homogenous smear layer, no open dentinal tubules

*Score 5*: a thick, homogenous smear layer covering the entire root canal wall

Score 1 and 2 were considered suitable scores (18). The data were statistically analyzed with one-way ANOVA test at a significance level of P<0.05.

**Figure 2:**
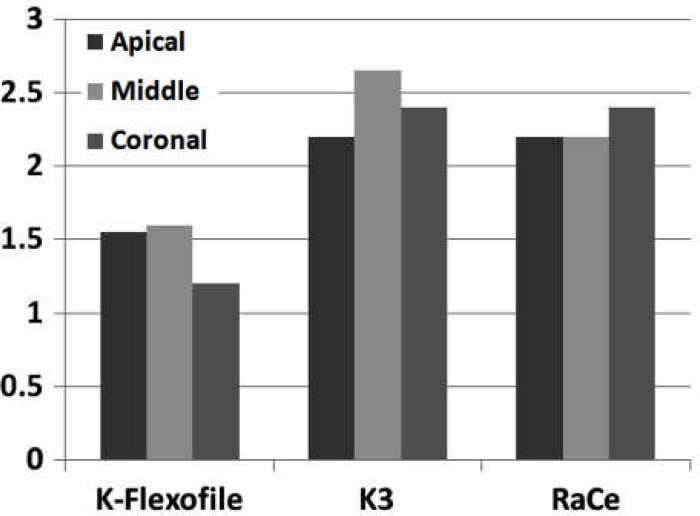
Debris comparison between the three groups in the three parts of the root canals

**Figure 3:**
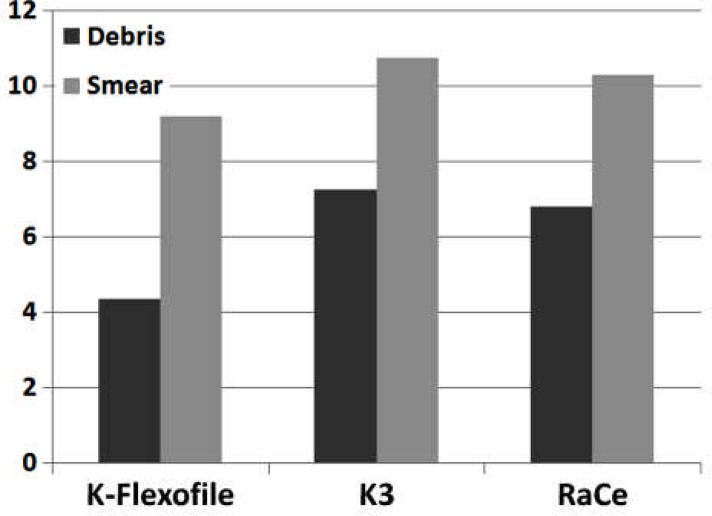
Smear layer and debris comparison between the three experimental groups throughout canals

## RESULTS

**Figure 4:**
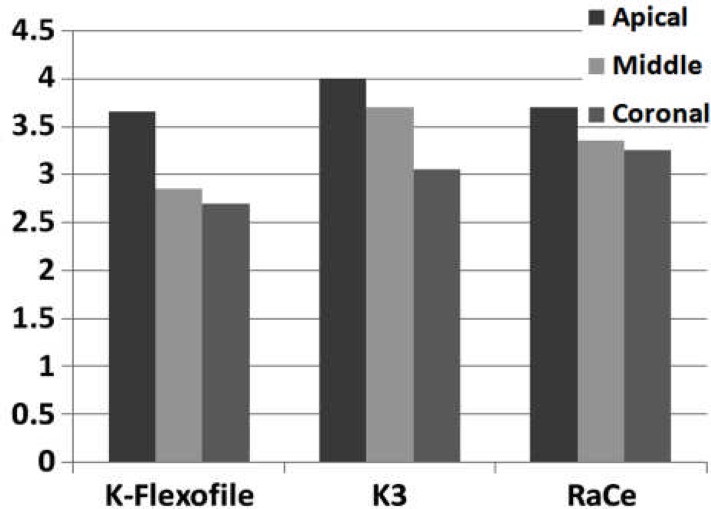
Smear layer comparison between the three groups in the three parts of the root canals

**Figure 5:**
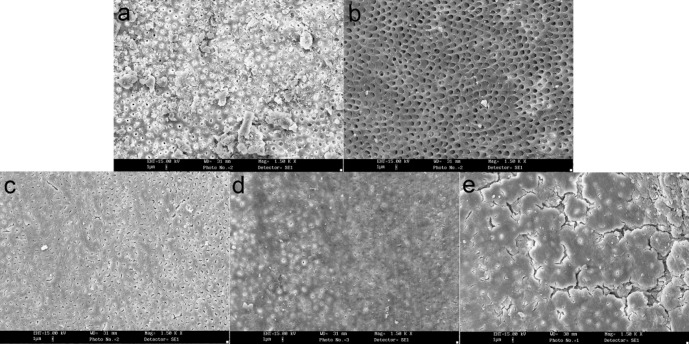
SEM images of smear layer remained on the canals after preparation (×1500 mag). (a) SEM image of the middle portion of the prepared canal with RaCe (Score 1),(b) SEM image of the middle portion of the prepared canal with RaCe (Score 2),(c) SEM image of the middle portion of the prepared canal with K-Flexofiles (Score 3),(d) SEM image of the coronal portion of the prepared canal with K-Flexofiles (Score 4),(e) SEM image of the apical portion of the prepared canal with K3 (Score 5).

Debris and smear layer were observed after instrumentation in all the groups and in all three parts of the root canal. Use of K-Flexofiles throughout the root canals resulted in less debris compared to K3 and RaCe rotary instruments (P<0.05) (Figure 1), (Figure 2) and (Figure 3). There were no significant differences between the three groups in smear layer removal on the root canal walls (Figure 3), (Figure 4) and (Figure 5). Also when comparing the amount of debris in the three parts of the root canal (coronal, middle and apical) significant differences were only found in the coronal parts of root canal walls; K-Flexofiles resulted in less debris compared to RaCe and K3 (P<0.05) (Figure 1), (Figure 2). Smear layer evaluation of the three root areas, interest- ingly found a significant difference in the middle part of the canals in which K-Flexofiles resulted in less smear compared to RaCe and K3 (P<0.05) (Figure 4), (Figure 5).

## DISCUSSION

Smear layer is created by the root canal preparation having a thickness of 1-2 µm (1,5). It is composed of mostly inorganic materials and is not found on uninstrumented areas (19). Although there are many controversy about effectiveness of smear layer removal in endodontic therapies, its removal seems desirable because it will increase dentin permeability, allowing better disinfection of deeper layers of the infected root canal dentin (13).

Debris is defined as dentin chips and residual vital or necrotic pulp tissue attached to the root canal walls, which is usually infected with bacteria (3). Thus, debris might prevent the efficient removal of bacteria from the root canal system. Also, debris may occupy part of the root canal space, which might also prevent complete obturation of the root canal (4).

In this study, cutting and cleaning efficacy of three instrumentation methods was examined on the basis of a separate numerical evaluation scheme for debris and smear layer (Hulsmann method) by means of SEM evaluation in the coronal, middle and apical portions of the canals (3). In previous studies, different magnifications ranging from ×15 to ×2500 were used (20,22). At low magnification large amounts of debris can easily be seen, but detail such as remnants of the smear layer or identification of dentinal tubules needs to be observed at higher magnifications. A disadvantage of using a higher magnification is the small size of the area of evaluation, potentially leading to misinterpretation (23).

Using the data of a pilot study in the present investigation, SEM evaluation was performed in ×500 and ×1500 magnifications for analysis of debris and smear layer (8).

To prevent discrepancy and bias in the results a key consideration is the consistency of the examiner’s evaluation and the blindness of the examiner to the various groups. , The samples in the present study were coded and randomly examined under SEM and the clinicians had no knowledge about the codes and the methods employed in preparation procedures.

In this study, partially uninstrumented areas with remaining debris and smear layer were found in all canal sections concurring with other studies (8). In general, the use of K-Flexofiles resulted in significantly less remnant debris compared to canal instrumentation with rotary K3 and RaCe instruments; these results corroborate with a previous report that showed hand K-Flexofiles to be superior in cleaning efficacy (9). The use of K-Flexofiles showed significantly less smear layer in middle part of canal compared to RaCe and K3. This finding was not in agreement with previous studies (9). Interestingly within the apical third no statistical difference was observed between the instrument groups. The clinical significance of this finding may have greater weight in endodontics, because the microorganisms which remain in the apical portion of the root canal are considered to be the main cause of treatment failure (24).

One of the reasons that may explain why hand K-Flexofiles show lower debris and smear layer scores than rotary RaCe and K3 instruments is the greater stiffness of K-Flexofiles; the greater force against the root canal wall may result in more efficient cleaning. In contrast, NiTi instruments used only in a rotary motion and without lingual and buccal pressure tend to only partially remove tooth structure. (23).

## CONCLUSION

K-Flexofiles resulted in less remnant debris within the root canal system compared to K3 and RaCe instruments. There were no significant differences between three groups with regards to smear layer removal in all three portions of the canal system.
